# Reconstruction of tensile and shear elastic moduli in anisotropic nearly incompressible media using Rayleigh wave phase and group velocities

**DOI:** 10.1117/1.JBO.30.12.124503

**Published:** 2025-08-05

**Authors:** Gabriel Regnault, Ruikang K. Wang, Matthew O’Donnell, Ivan Pelivanov

**Affiliations:** a Univ Lyon, Université Claude Bernard Lyon 1, Centre Léon Bérard, INSERM, UMR, LabTAU, Lyon, France; b University of Washington, Department of Bioengineering, Seattle, Washington, United States

**Keywords:** optical coherence elastography, elastic moduli of soft tissues, Rayleigh waves, phase velocity, group velocity, nearly-incompressible transverse isotropic

## Abstract

**Significance:**

Dynamic optical coherence elastography can excite and detect propagating mechanical waves in soft tissue without physical contact and in near real time. However, most soft tissue is anisotropic, characterized by at least three independent elastic moduli. As a result, reconstructing these moduli from mechanical wave fields requires a complex procedure.

**Aim:**

We consider a nearly incompressible transverse isotropic (NITI) material, which has been shown to locally define the symmetry of many soft tissues such as muscle, tendon, skin, cornea, heart, and brain. Reconstruction of elastic moduli in the NITI medium using Rayleigh waves is addressed here. A method to accurately compute the angular dependence of Rayleigh wave phase velocity for the most common geometries (point-like and line sources) of mechanical wave excitation is described.

**Approach:**

When a line source is used to launch plane mechanical waves over the medium surface, the phase velocity of Rayleigh waves in the direction of propagation is directly accessible. For a point-like source, propagation of the energy flux is tracked (i.e., its group velocity), which cannot be directly used for moduli inversion. In this case, angular spectrum decomposition is used to access the phase velocity. Both numerical simulations in OnScale and experiments in a stretched PVA phantom were performed.

**Results:**

We show that both methods (line source wave excitation and angular decomposition from a point-like source) produce similar results and accurately estimate the angular anisotropy of the Rayleigh wave phase velocity. We also explicitly show that a commonly used group velocity approach leads to inadequate moduli inversion and should not be used for reconstruction.

**Conclusions:**

We suggest that the line source is best when a surface area must be scanned, whereas the point-like source with the proposed phase velocity reconstruction is best for single-point moduli estimation or when tissue motion is a concern.

## 1 Introduction

Optical coherence elastography (OCE) is an effective method to probe deformational properties of soft tissue.^1^^,^^2^ As in magnetic resonance (MR)- and ultrasound (US)-based methods, both static and dynamic approaches can be used. Static OCE usually measures strain induced in tissue by an external load. Although a very clear strain contrast can be observed for tissues with different Young’s moduli, quantitative reconstruction of tissue elastic moduli is problematic due to typically unknown stresses during *in vivo* studies.^3^ In addition, contact loads may not be acceptable in many medical applications, such as ophthalmology and dermatology.

Dynamic OCE uses propagating mechanical waves.^2^^,^^4^ As shear waves in soft tissue are slow (typically a few meters per second), they can be tracked in space and time with phase-sensitive optical coherence tomography (PhS-OCT). The A-scan rate of current Fourier-domain OCT systems ranges between tens of kHz to a few MHz, making it possible to probe kHz-rate propagating mechanical waves. The wave speed is fully defined by tissue elasticity and, therefore, elastic moduli can be reconstructed from the OCE-measured wave speed.

The latest generation of dynamic OCE systems is robust,^5^^,^^6^ where they use noncontact excitation of mechanical waves from air with reflection-based, air-coupled acoustic radiation force [also called as acoustic micro-tapping (AμT)]. The acoustic power coupled into tissue for AμT is much below medical limits. As propagating waves are detected by FDA-approved OCT systems satisfying all medical requirements, dynamic OCE is completely noncontact and noninvasive.

The matrix of elasticity, C, for an isotropic medium can be characterized by two independent elastic constants (Lamé constants) λ and μ:

$$C=[\begin{array}{cccccc} \lambda +2\mu & \lambda & \lambda & & & \\ \lambda & \lambda +2\mu & \lambda & & & \\ \lambda & \lambda & \lambda +2\mu & & & \\ & & & \mu & & \\ & & & & \mu & \\ & & & & & \mu \end{array}].$$ (1)

A typical soft biological tissue is nearly incompressible, which means that its shear modulus μ is many orders of magnitude smaller than the bulk modulus $K=\lambda +\frac{2}{3}\mu$ (i.e., λ≫μ). This results in a simple expression for the Young’s modulus E, which is solely defined by the shear modulus^7^^,^^8^

E=3μ. (2)

The speed of propagating shear waves is equal to $c_{S}=\sqrt{\mu /\rho }$. Thus, tissue Young’s modulus, and therefore its tensile properties, can be simply computed as

$$E=3\rho c_{s}^{2}.$$ (3)

This expression is used by most US and MR scanners to compute tissue bulk properties (ρ is material density).

As in many publications, we did not specify “phase” in the definition of $c_{S}$. Indeed, in most theoretical solutions of Christoffel’s equation defining wave propagation in an elastic medium, the plane wave solution is usually considered.^9^^,^^10^ In this approximation, if tissue phase velocity frequency dispersion due to viscosity can be neglected, the phase velocity of mechanical waves is equal to the group velocity. However, in practical applications of OCE, the source of mechanical waves is usually localized, making the plane-wave solution in the near field inaccurate because of additional evanescent modes excited near the source. For an accurate description of wave propagation close to the source, a Green’s function approach should be used.^11^^–^^13^

Furthermore, many soft biological tissues are not isotropic - organs often contain collagen fibers aligned in a certain way to optimize their deformational properties for a particular function, which induces local tissue anisotropy. Thus, a realistic elastic model can be quite complicated. For example, a model of a nearly incompressible transverse isotropic (or NITI^14^^–^^17^) medium, in which a symmetry axis defines a preferred local fiber orientation, covers a broad spectrum of tissues and organs, such as skin, muscle, tendon, heart, and cornea. In a NITI medium, expressions (1 to 3) are not adequate because there are three independent shear moduli, implying a directional dependence of the wave propagation velocity relative to the symmetry axis (collagen fiber orientation).^14^^–^^17^ A careful measurement of the angular anisotropy of the phase velocity defines the accuracy with which all three mechanical moduli can be reconstructed.^15^^–^^17^

In addition to the near-field effects of wave propagation mentioned above,^13^ anisotropic media introduce another serious challenge—spatial dispersion of mechanical waves^17^ due to variations in wave phase (defining phase velocity) between propagation directions and wave energy (associated with group velocity). This phenomenon can lead to effects such as group velocity focusing,^18^^,^^19^ where the wave energy becomes localized in certain directions, or ambiguities in group velocity measurements (see Sec. 2). Angular dependencies of phase and group velocities are very different in anisotropic media, and fitting the measured group velocity anisotropy with the analytical solutions obtained for the phase velocity is fundamentally incorrect.

This paper describes the spatial dispersion of Rayleigh waves in anisotropic soft media, a typical situation for dynamic OCE. We consider the most common geometries of mechanical wave excitation—point-like and line sources—and analytically and experimentally show how to correctly treat measured velocity spatial dispersion to reconstruct elastic moduli in anisotropic soft tissue, with a particular focus on the NITI medium.

## 2 Method

### 2.1 NITI Model

We will consider a NITI medium as the most common model of local anisotropy within biological tissue, where we assume that collagen fibers locally have a preferred direction coinciding with the Z-axis.

The matrix of elasticity of a NITI medium is described by four independent elastic constants:^15^

$$C=[\begin{array}{cccccc} \lambda +2\mu & \lambda & \lambda & & & \\ \lambda & \lambda +2\mu & \lambda & & & \\ \lambda & \lambda & \lambda +2\mu +\delta & & & \\ & & & G & & \\ & & & & G & \\ & & & & & \mu \end{array}]\;\;,$$ (4)

where λ and μ are the conventional Lamé constants. An additional modulus G shows that shear deformation can be different if shear stress is applied along the symmetry axis z compared with that applied across it; and δ characterizes the NITI medium tensile anisotropy. Again, λ is much larger than any other moduli and does not affect Young’s moduli and Poisson’s ratios.

The main deformational properties of the NITI medium, the Young’s moduli, and Poisson’s ratios can be derived from Eq. (4)^15^

$$E_{T}=3\mu +\mu [\frac{\delta }{4\mu +\delta }],\;E_{L}=3\mu +\delta ,\;\nu_{TT}=\frac{1}{2}[1+\frac{\delta }{4\mu +\delta }]=1-\frac{1}{2}\frac{E_{T}}{E_{L}},\;\nu_{TL}=\frac{1}{2}[1-\frac{\delta }{4\mu +\delta }]=\frac{1}{2}\frac{E_{T}}{E_{L}},\;\nu_{LT}=\frac{1}{2}.$$ (5)

Here, $E_{L,T}$ are the Young’s moduli, determining tensile properties, in the direction along and perpendicular to the symmetry axis, respectively. The Poisson’s ratio $\nu_{LT}=1/2$ characterizes a deformation in the isotropy plane when the stress is applied along the symmetry axis. The other two Poisson’s ratios $\nu_{TL}$ and $\nu_{TT}$ characterize tissue deformation along and across the symmetry axis when the stress is applied perpendicular to the axis.

We note a few important conclusions from Eq. (5). (i) Compared with the isotropic case, there are two parameters, μ and an additional parameter δ, that define Young’s moduli and Poisson’s ratios. (ii) All Young’s moduli and Poisson’s ratios do not depend on the shear modulus G. (iii) The fact that $\nu_{LT}$ equals 1/2 means that the deformation will be distributed equally in the isotropy plane when the stress is applied along the symmetry axis. (iv) When the stress is applied perpendicularly to the fiber direction, however, the deformation will be distributed unequally along and perpendicular to the fibers, but the sum of their Poisson’s ratios must equal unity:

$$\nu_{TT}+\nu_{TL}=1.$$ (6)

### 2.2 Phase and Group Velocities

In a NITI material, an analytical solution for the Rayleigh wave speed is not readily available. Consequently, numerical methods such as Stroh Formalism must be used.^20^ This approach provides the phase velocity of plane waves for any propagation direction relative to the symmetry axis as a function of elastic moduli μ, G, and δ.

In an anisotropic medium, in general, the phase velocity $c_{ph}$ of wave propagation is equal to the group velocity $c_{g}$ only along principal axes, whereas for other propagation directions, they are connected by^21^

$$c_{g}=c_{ph}\sqrt{1+{\left(\frac{1}{c_{ph}}\frac{dc_{ph}}{d\theta }\right)}^{2}+\frac{1}{\sin^{2}\;\theta }{\left(\frac{1}{c_{ph}}\frac{dc_{ph}}{d\varphi }\right)}^{2}}\;\;,\;\;$$ (7)

$$\frac{1}{c_{ph}}=\frac{1}{c_{g}}\sqrt{1+(\frac{1}{c_{g}}\frac{dc_{g}}{d\theta_{g}}{)}^{2}+\frac{1}{\sin^{2}\;\theta_{g}}(\frac{1}{c_{g}}\frac{dc_{g}}{d\varphi_{g}}{)}^{2}},$$ (8)

where θ and φ, and $\theta_{g}$ and $\varphi_{g}$ are polar and azimuth angles for phase and energy flux, respectively.

In a NITI material, Eqs. (7) and (8) are simplified to

$$c_{g}=c_{ph}\sqrt{1+(\frac{1}{c_{ph}}\frac{dc_{ph}}{d\theta }{)}^{2}},$$ (9)

$$\frac{1}{c_{ph}}=\frac{1}{c_{g}}\sqrt{1+(\frac{1}{c_{g}}\frac{dc_{g}}{d\theta_{g}}{)}^{2}}.$$ (10)

The propagation direction of the wave energy flux $\theta_{g}$ differs from that of the wave phase θ

$$\theta_{g}=\theta +\arctan(\frac{1}{c_{ph}}\frac{dc_{ph}}{d\theta }),$$ (11)

Although Eqs. (9)–(11) can be used to convert the group velocity angular dependence into the phase velocity angular dependence, its implementation in practice is not robust due to the presence of derivatives requiring highly oversampled angular measurements. In addition, tissue heterogeneities may make the group-to-phase velocity conversion highly inaccurate, again, due to the derivative term.

Below, we show how phase velocity angular dependence, required for elastic moduli inversion, can be obtained for different experimental configurations.

### 2.3 Numerical Simulations

We developed a finite element numerical model of mechanical wave propagation in a NITI material using OnScale (OnScale, Redwood City, California, United States). The computational domain was discretized using linear finite elements on a regular rectangular grid with at least 40 elements per elastic wavelength. Simulations were performed using explicit time stepping, and a vertical vibration velocity component was extracted for analysis to match OCE experiments. In simulations, we used two excitation geometries—a point-like source and a line source—representing the most common ones for dynamic OCE.^5^^,^^6^ We used simulations to perform a parametric study on elastic properties of nonviscous NITI media with a fixed value of μ=10  kPa, but different shear and tensile anisotropy ratios: $\frac{G}{\mu }=[1,2,4]$ and $\frac{\delta }{\mu }=[0,2,4]$. The range of parameters was chosen based on our prior *in vivo* skin studies.^15^

#### 2.3.1 Point source

**Fig. 1:**
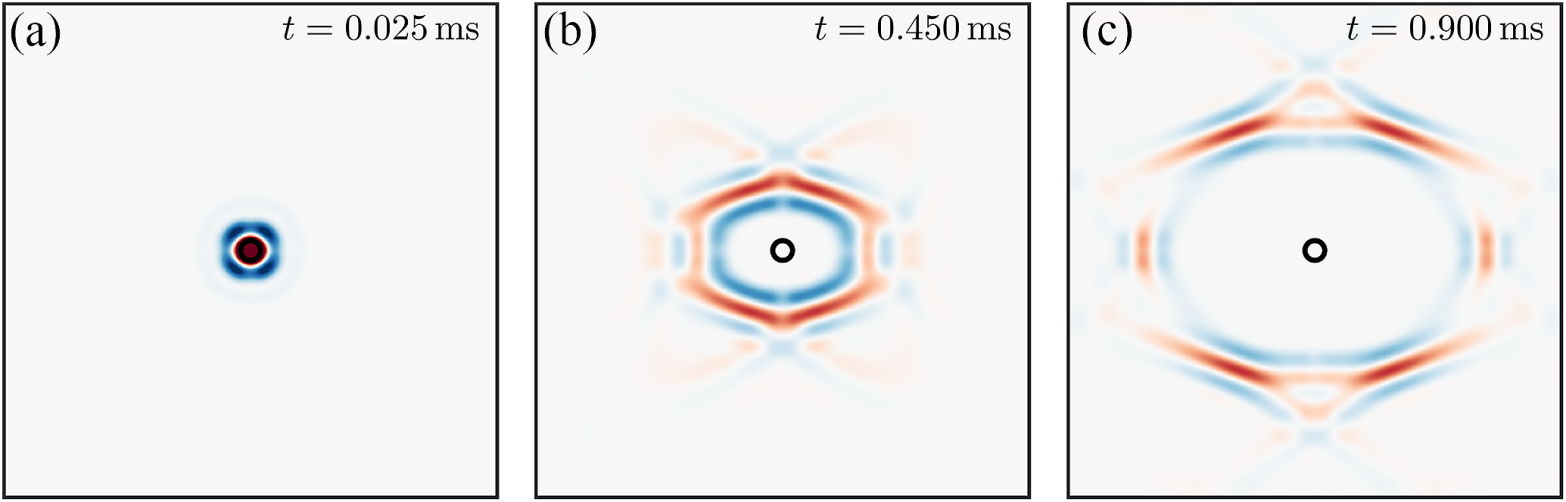
Wavefields generated by a point-like source at different time moments from the excitation: (a) t=0.025  ms⁡, (b) t=0.450  ms⁡, and (c) t=0.900  ms⁡. For this case, μ=10  kPa, G/μ=4, δ/μ=0. Note, that $E_{L}$ and $E_{T}$ are the same for this case (i.e., there is no tensile anisotropy) (Video 1, MP4, 6.16 MB [URL: https://doi.org/10.1117/1.JBO.30.12.124503.s1]).

To reduce computation time, only one quarter (from 0 to 90 deg) of the angular domain was analyzed for the point source with symmetry conditions at the boundaries. The full 3D field was then combined from four quadrants (Fig. 1). The total field of view was 10×10  mm. The thickness of the material was sufficient to avoid wave guidance for the frequency range considered (h=3  mm, f∈[0−5]  kHz).

OCE detects the distribution of the magnitude of the propagating Rayleigh wave packet, which propagates radially away from the source with a group velocity. The propagation is characterized by heterogeneity in the signal magnitude for different propagation directions, group velocity focusing points, and ambiguities in some propagation directions. This behavior is typical for anisotropic media. As shown below, the true phase velocity can be very different from the group velocity directly computed from the wavefields of Fig. 1. A video of wave propagation following point-source excitation for the case shown in Fig. 1 is provided in Video 1.

#### 2.3.2 Line Source

**Fig. 2:**
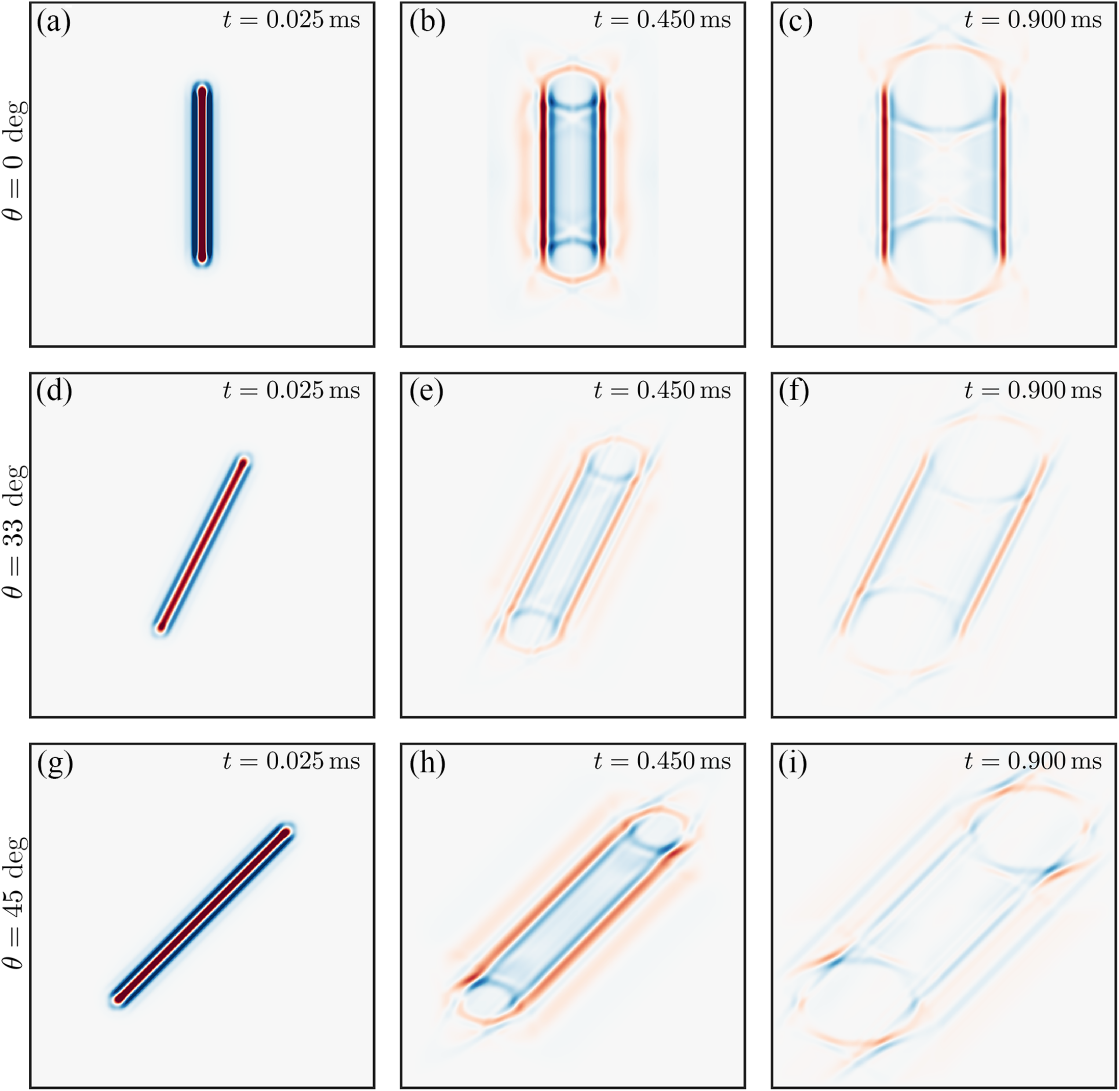
Wavefields generated by a line source at different propagation directions characterized by angle θ relative to the symmetry axis Z at different time moments from the excitation. For this particular case, μ=10  kPa, G/μ=4, δ/μ=0. Young’s moduli $E_{L}$ and $E_{T}$ are the same for this case (i.e., there is no tensile anisotropy). Panels (a)–(c): θ=0  deg⁡, time instants from the excitation are t=0.025  ms⁡, t=0.45  ms⁡, and t=0.9  ms⁡, respectively. Panels (d)–(f): θ=33  deg⁡, time instants from the excitation are t=0.025  ms⁡, t=0.45  ms⁡, and t=0.9  ms⁡, respectively. Panels (g)–(i): θ=45  deg⁡, time instants from the excitation are t=0.025  ms⁡, t=0.45  ms⁡ and t=0.9  ms⁡, respectively (Video 2, MP4, 6.11 MB [URL: https://doi.org/10.1117/1.JBO.30.12.124503.s2]).

We also simulated line source excitation, which was used previously in many experimental dynamic OCE studies.^4^^,^^5^^,^^13^^–^^15^^,^^22^^–^^26^ It does not require additional simulations in OnScale. Instead, using the wave field generated by the point source, the line source was obtained by superposition of point-like sources using the Huygens–Fresnel principle with a source density equal to 100 points per millimeter. Different propagation directions in the NITI material for the line source were obtained by rotating the coordinate system, as illustrated in Fig. 2.

As the length of the line source was much larger compared with its width, diffraction effects could be ignored in the center of the beam, and hence, wave propagation for distances smaller than the source length (in the near field of the line source) could be considered plane-wave propagation with a speed expected to be equal to the phase velocity. Like that for the point source, the magnitude of the propagation signal strongly depends on the propagation direction. A video of wave propagation following multiple line source excitation for the case shown in Fig. 2 is provided in Video 2.

## 3 Experiment

### 3.1 Anisotropic PVA Phantom

An anisotropic polyvinyl alcohol (PVA, 146-186 kDa, >99% hydrolyzed, CAS: 9002-89-5, Sigma-Aldrich, St. Louis, Missouri, United States) phantom was made using a specific freeze-thaw sequence to induce NITI symmetry.^27^ In detail, 6% wt. PVA was dissolved in water at 90°C for 1 h. Once complete dissolution was achieved, a 0.1% ${\mathrm{TiO}}_{2}$ wt. solution was added for optical contrast. When completely mixed, the solution was degassed and poured into a 10×5×4  cm rectangular mold. The solution underwent three freeze-thaw cycles (cooling down to −20°C and then warming up to room temperature). The first cycle polymerized the phantom and gave it a solid but elastic structure. Then, after thawing, both ends of the phantom were clamped, and a 10 cm stretch was applied in the longer length direction. Immediately after stretching, a second freeze-thaw cycle was performed. Then, the second 10 cm stretch was applied before the last freeze-thaw cycle. Before completing final thawing, about a millimeter-thick layer of a phantom was removed from the surface with a sharp razor blade to obtain a flat interface and thus improve wave detection with OCE. This process produced a NITI material with the symmetry axis along the stretch direction. Only the central part of the phantom (∼5×5×2  cm in dimensions) was used for experiments.

### 3.2 Experimental Setup

Only plane wave excitation was used for the experiments. A cylindrically focused air-coupled ultrasound transducer operating at its resonant 1.05 MHz central frequency was the AμT source. The transducer was made from a 75-deg and 11 mm wide segment of a piezo-ceramic tube (part # 42-1052)^28^ with a matching layer^5^ attached to its surface for efficient ultrasound transmission from ceramic to air. Transducer orientation and location were adjusted to match its focal plane with the phantom interface. It introduced localized, reflection-based acoustic radiation force at the sample interface, producing a “line” load to the sample surface. The focal spot was 11 mm in length and ∼0.5  mm in width. A short, 100  μs duration, pulse generated broadband mechanical waves. Displacement relaxation within the phantom excited quasi-planar broadband wave packets (in skin,^15^ typically up to 4 kHz) of Rayleigh, evanescent (or SEW^13^), and oblique (bulk) quasi-shear mechanical waves inside the medium. Detailed information about the AμT transducer and its operating principle can be found in Refs. 5, 13, and 29.

Elastic waves were tracked at the phantom surface using a spectral domain phase-sensitive OCT system operating at a 46.5 kHz A-line rate.^30^ M-B mode wave-tracking was used: for every tracking position along a 10 mm scanning range (256 positions stepped at $d_{x}=54.7\;\;\mu m$), one AμT push was sent, and 200 consecutive A-lines were recorded. With the depth encoding of OCT (512 pixels in depth, $d_{z}=5\;\;\mu m$), a 3D spatio-temporal image was formed. For every propagation angle, the scanning axis matched the wave propagation direction (orthogonal to the AμT line source). M-B datasets were then used to obtain propagating mechanical wave time-distance profiles. The local vertical vibration velocity, $\Delta v_{z}(x,z,t)$, was computed using the optical phase difference between two consecutive A-lines (Δφ(x,z,t)) for every lateral and axial position

$$\Delta v_{z}(x,z,t)=\frac{\Delta \varphi (x,z,t)\overset{¯}{\lambda }f_{s}}{4\pi \overset{¯}{n}},$$ (12)

where $\overset{¯}{\lambda }=1310\;\;\mathrm{nm}$ is the central wavelength of the broadband source (∼46  nm spectral bandwidth), $f_{s}$ is the sampling frequency, and $\overset{¯}{n}=1.3$ is the optical index of the phantom, assuming negligible effects of PVA and ${\mathrm{TiO}}_{2}$. After surface segmentation, the Rayleigh wave spatio-temporal displacement maps were obtained by averaging $\Delta v_{z}$ over the first 20 pixels below the phantom interface to improve the signal-to-noise ratio.

The phantom was mounted on a rotating platform for angular wave propagation measurements. Propagation directions between 0 and 180 deg were probed, with a 10 deg increment. The phantom was aligned such that the symmetry axis (direction of stretching) corresponded to θ=0  deg. For each propagation direction, three scans were taken for statistical analysis.

## 4 Results

### 4.1 Results of Numerical Simulations

#### 4.1.1 Comparison of point-like and line sources

**Fig. 3:**
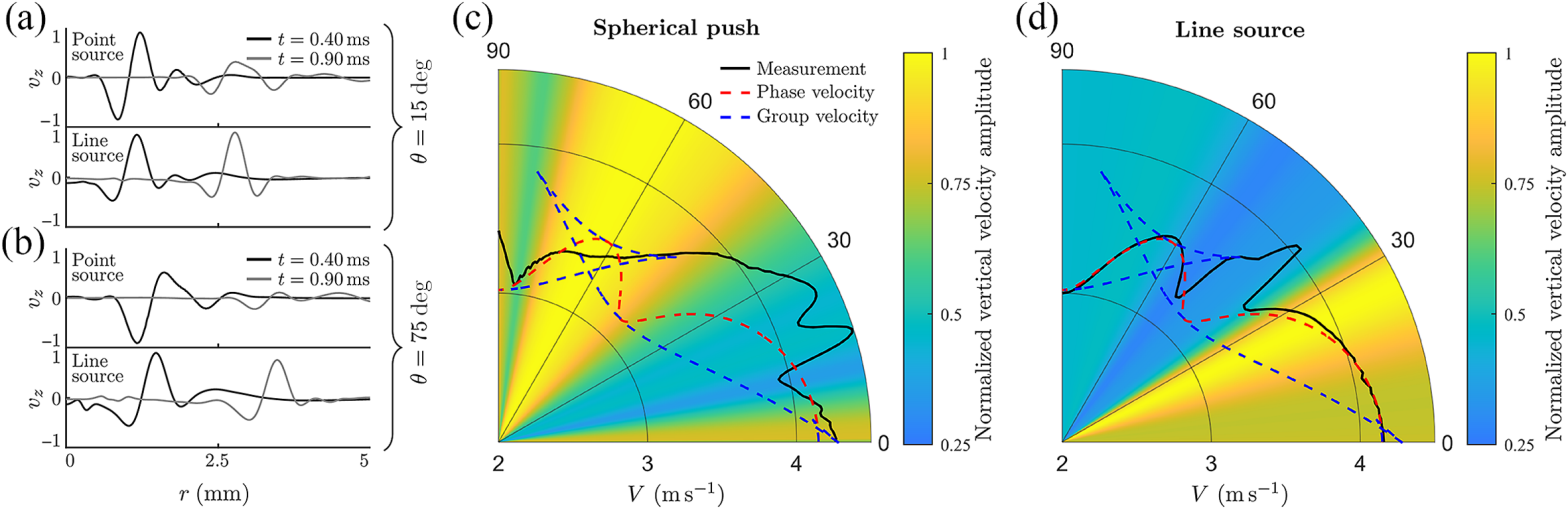
Comparison of Rayleigh wave velocities computed from numerically simulated (OnScale) wave propagation using a correlation method^30^ for both point-like and line sources of Rayleigh wave excitation. NITI medium mechanical moduli are the same as in Figs. 1 and 2: μ=10  kPa, G/μ=4, δ/μ=0 (significant shear anisotropy but no tensile anisotropy). (a) Comparison of the spatial signal profiles for point-like and line sources for two time moments ($t_{1}=0.4\;\;\mathrm{ms}$ and $t_{2}=0.9\;\;\mathrm{ms}$) in the direction of (a) θ=15  deg and (b) θ=75  deg relative to the symmetry axis. Comparison of the theoretically calculated group (dashed blue line) and phase (dashed red line) velocities and the measured Rayleigh wave speed using cross-correlation for point source (c) and line source (d) excitation. The colormap corresponds to the amplitude of the wave at t=0.9  ms, computed as the integral of the absolute value of the wavefields shown in (a) and (b) for every direction.

Using the wave-fields shown in Figs. 1 and 2 and Videos 1 and 2 in the Supplementary Material, we computed the propagation speed using the correlation method^30^ for both excitation geometries. For better precision, the wavefields in every direction were upsampled by a factor of 100. Figure 3 shows detailed results for a NITI material with μ=10  kPa, G/μ=4, and δ/μ=0 (i.e., significant shear anisotropy but no tensile anisotropy).

Again, for a point source, the computed velocity should equal the group velocity. By contrast, for the line source propagation, speed should equal the phase velocity because it creates plane Rayleigh waves.

Figures 3(a) and 3(b) show how different Rayleigh wave shapes can depend on the propagation direction. For the line source, shape changes mainly produce variations in the signal magnitude, whereas for the point source propagation, signals change in both shape and magnitude (see Video 1, in particular, in the 90 deg orientation, where the wave splits after t=0.5  ms). The angular distribution of the signal magnitude is shown in the background of Figs. 3(c) and 3(d) using a hot scale [the amplitude is computed for every angle at the same time instant t=0.9  ms, as the integral of the absolute value of the wavefields shown in Figs. 3(a) and 3(b)]. Note that the conditions of maximum signal magnitude for the point source may produce the minimum signal magnitude for the plane wave created by the line source in the same direction (θ≈45  deg, for example, see Video 2).

Figures 3(c) and 3(d) indicate how different the propagation speeds can be with different excitation geometries. Note that the theoretical phase and group velocities were computed using the Stroh formalism^15^ and Eq. (9). For a point source [Fig. 3(c)], the maximum signal magnitude is observed for the range of angles (between 45 and 75 deg) where focusing of the group velocity is predicted in theory (dashed blue curve). Over this range of directions, the group velocity is ambiguous and cannot be computed correctly. This ambiguity also greatly affects other propagation directions, making it very inaccurate to compute even the group velocity. Thus, the computed group velocity (black curve) is very different from the theoretically predicted (dashed blue line). It is also very different from the phase velocity (dashed red curve) used in all equations for elastic moduli reconstruction. In addition, for realistic viscoelastic materials, due to the broad frequency spectrum of Rayleigh wave signals, the computed group velocity will also depend on frequency content. This makes group velocity measurements very difficult and inaccurate for computing the mechanical moduli of anisotropic soft tissues.

The effect observed above can be understood if we consider the wave’s spatial spectrum. When a line source is used, it generates a plane wave in the near field of the source. Its spatial (or angular) spectrum is a delta function because the direction of propagation is defined and detection is performed along this direction. This avoids the superposition of different angular spectral harmonics, and therefore, the phase velocity will characterize wave propagation in the excitation direction.

By contrast, the spatial spectrum of the point source is equally distributed over propagation angles. Therefore, each point of the wavefront is a superposition of waves arriving from different directions, resulting in a group velocity of wave packet propagation. Because of the broad bandwidth nature of OCE signals, the problem is even more complicated because the mixture of time harmonics also contributes. Without proper unmixing, the measured group velocity cannot be used to reconstruct tissue elastic moduli. We emphasize this point here because, unfortunately, many studies ignore these effects or treat measured characteristics incorrectly.

**Fig. 4:**
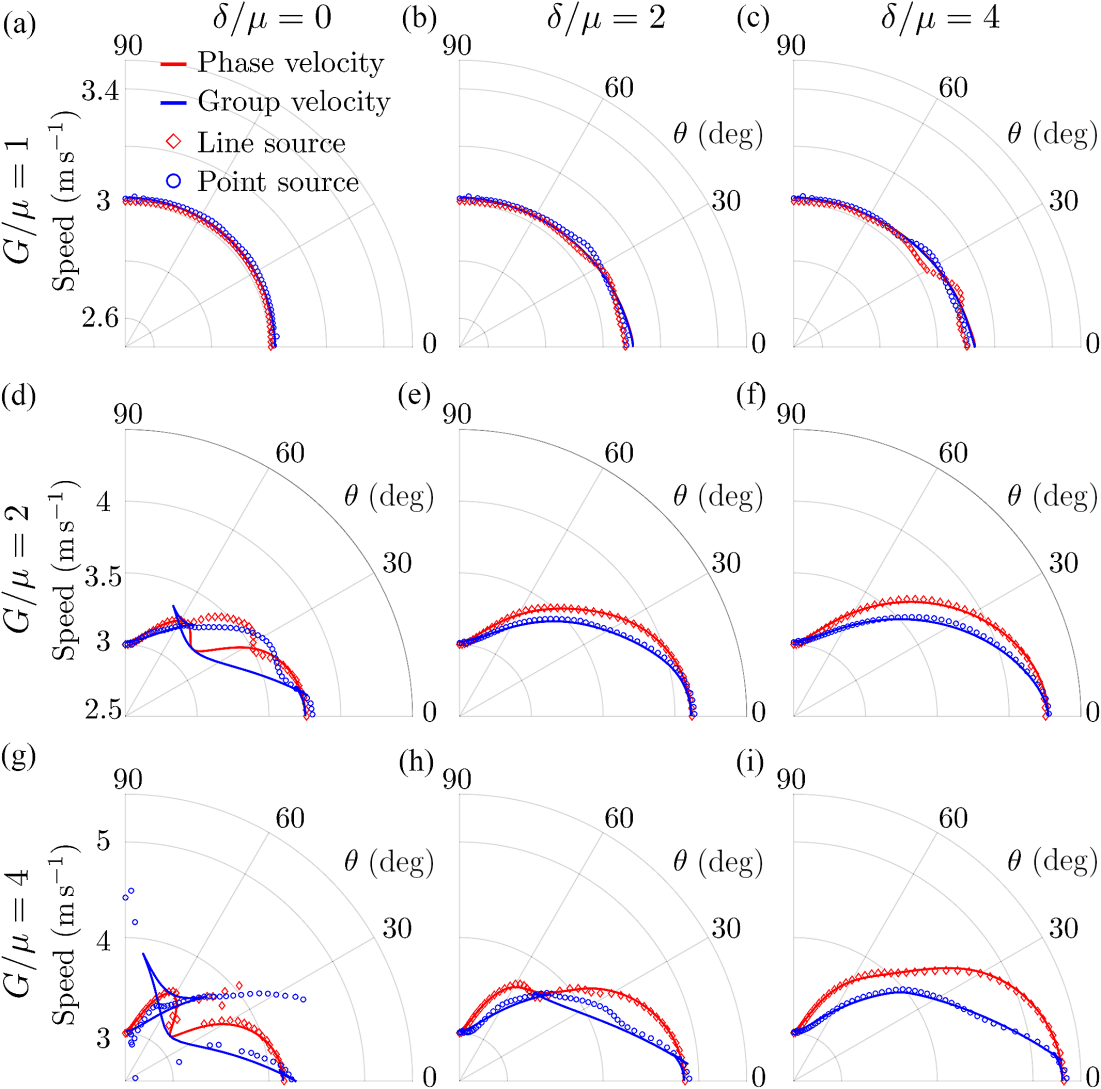
Comparison of Rayleigh wave velocities computed from numerically simulated (OnScale) wave propagation using a correlation method^30^ for both point-like and line sources for a broad range of NITI medium mechanical properties: (a) G/μ=1, δ/μ=0, (b) G/μ=1, δ/μ=2, (c) G/μ=1, δ/μ=4, (d) G/μ=2, δ/μ=0, (e) G/μ=2, δ/μ=2, (f) G/μ=2 δ/μ=4, (g) G/μ=4, δ/μ=0, (h) G/μ=4, δ/μ=2, (i) G/μ=4, δ/μ=4. Solid curves correspond to ground truth values of phase (red curve) and group (blue curve) velocities of Rayleigh waves computed analytically.

Computed phase and group velocities of Rayleigh waves are compared with those measured based on simulated wave profiles for a broad range of relationships among μ, G, and δ in Fig. 4. This figure shows that the group velocity computed from wave propagation originating from the point-like source correctly reflects medium properties only for the isotropic case (G/μ=1, δ/μ=0). In most situations, the measured group velocity does not equal either the phase velocity or even its theoretical value. The group velocity value extracted from simulations corresponded well to theoretical predictions for the case of very strong tensile and shear anisotropies (G/μ=4, δ/μ=4).

By contrast, the propagation speed of Rayleigh waves originating from the line source matches theoretical predictions very well, except in a few regions of very small signal magnitude. This clearly indicates that the computation of phase velocity is required for the correct reconstruction of mechanical properties from dynamic OCE measurements in anisotropic tissue.

#### 4.1.2 Computation of the phase velocity of the Rayleigh wave launched by a point-like source

In Sec. 4.1.1, we clearly showed that, for point-like excitation of Rayleigh waves, the computed propagation wave speed angular distribution is different not only from the phase velocity angular distribution but also from the theoretically predicted group velocity due to a complicated superposition of different temporal and spatial harmonics of the wavefield in a NITI media. The question is if the phase velocity angular distribution can still be extracted from OCE measurements when point-like excitation is employed because it has been applied routinely by many research groups. This section describes step-by-step how it can be done.

**Fig. 5:**
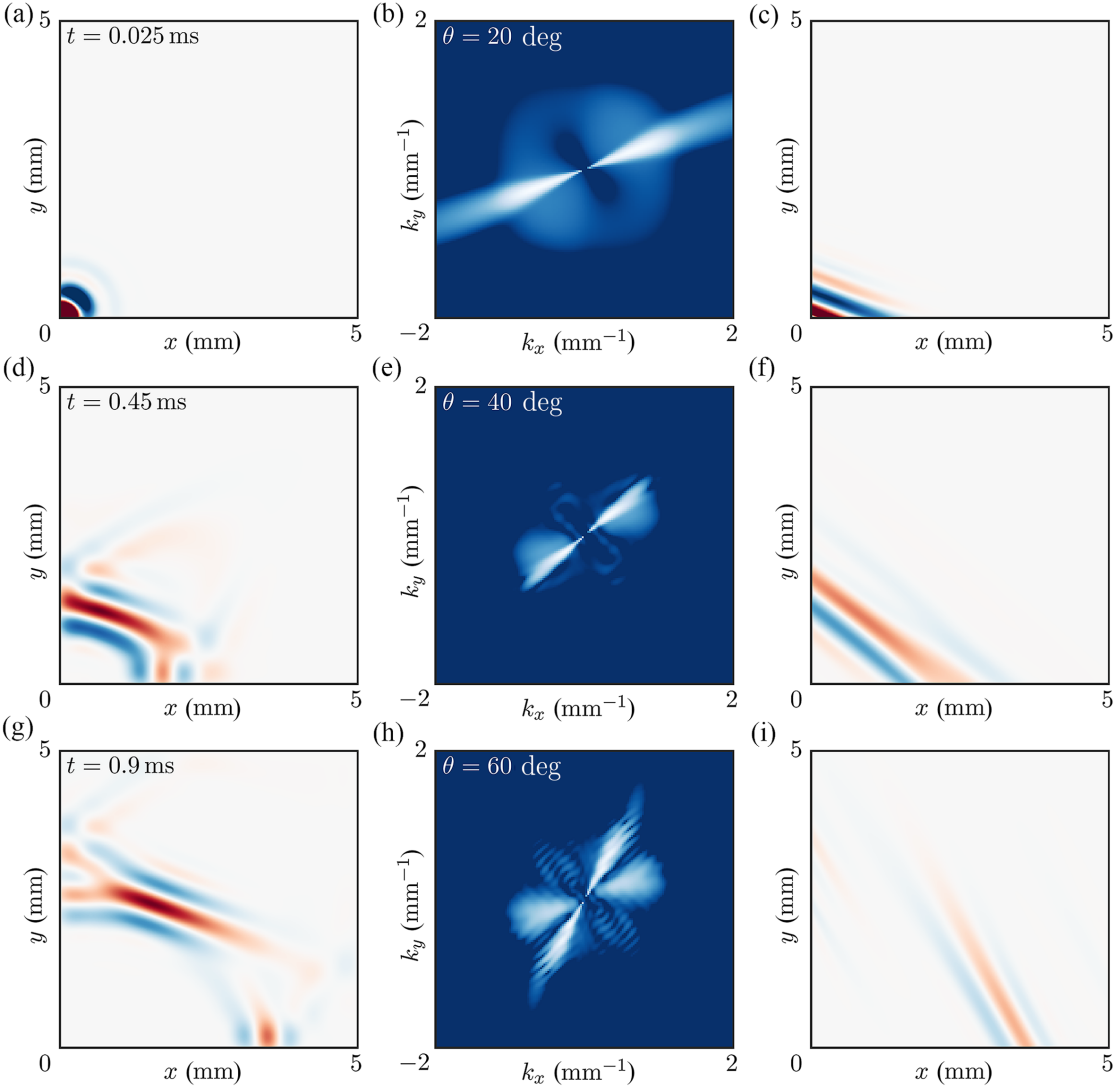
Angular decomposition method to compute the phase velocity of Rayleigh waves launched by a point-like source in a NITI medium. NITI medium mechanical moduli are the same as in Figs. 1 and 2: μ=10  kPa, G/μ=4, δ/μ=0 (i.e., significant shear anisotropy but no tensile anisotropy). (a), (d), (g) Wavefields originating from the point-like source at different time instants as indicated in the top left corner of the panels. (b), (e), (h) 2D spatial spectra computed for the wavefields (a), (d), (g) respectively and their filtration with a 5 deg width Gaussian angular filter in the propagation direction indicated in the top left corner of each spectral panel. (c), (f), (i) – filtered waveforms for the same time instants (a), (d), (g) and directions (b), (e), (h).

As mentioned above, the main reason for the highly complex nature of the phase velocity angular distribution is the superposition of different spatial harmonics of the signal spectrum. Thus, the key to suppressing these effects is spectral unmixing, which can be done in k-space. Figure 5 shows how wavefields [Figs. 5(a), 5(d), and 5(g)] recorded at an arbitrary time moment can be processed. The idea is to filter out all spatial harmonics of the spectrum except the one corresponding to propagation in the desired direction [Figs. 5(b), 5(e), and 5(h)]. Applying the inverse Fourier Transform to the filtered spectrum, the wavefield of a plane wave propagating in a desired direction can be obtained [Figs. 5(c), 5(f), and 5(i)]. Clearly, processed wavefields differ greatly from the original unfiltered wavefields. To obtain these results, we used a Gaussian filter with a 5 deg width at the 1/e level. This width can be further optimized based on experimental conditions, the expected level of anisotropy, signal-to-noise ratio, and other factors. It can also be more aggressive, such as a super-Gaussian for example. However, increasing the width of the spatial filter should be done with care as it can lead to additional angular mixing and eventually corrupt the phase velocity estimate. Depending on the application, it is essential to analyze the angular spectrum before adapting the filter as its width depends on the degree of material anisotropy. The higher the anisotropy, the narrower the filter should be.

**Fig. 6:**
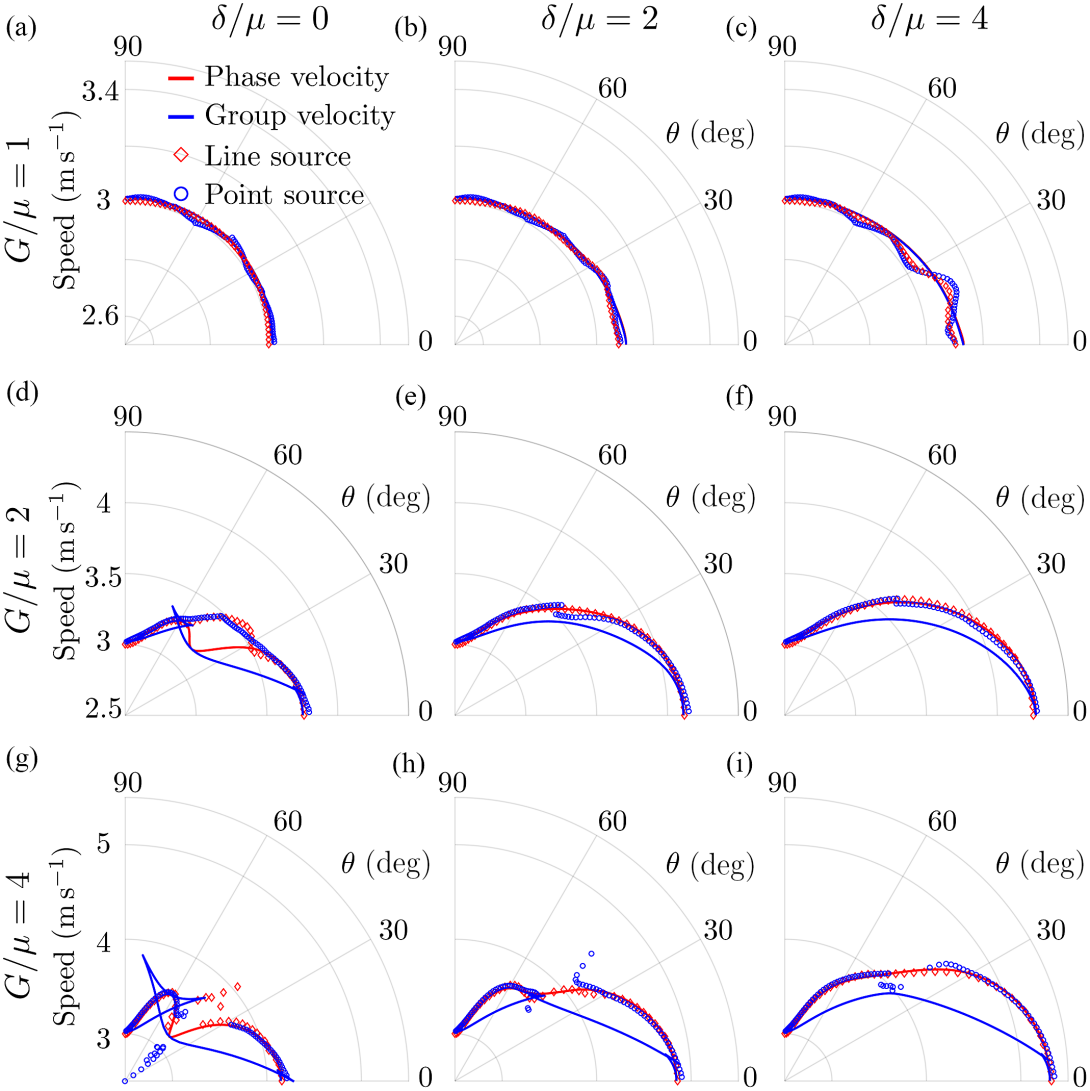
Rayleigh wave phase velocity angular dependence (blue dotted curve) obtained by spatial filtration (described in Sec. 4.1.2) of wavefields originating from the point-like source for a broad range of NITI medium mechanical properties: (a) G/μ=1, δ/μ=0, (b) G/μ=1, δ/μ=2, (c) G/μ=1, δ/μ=4, (d) G/μ=2, δ/μ=0, (e) G/μ=2, δ/μ=2, (f) G/μ=2, δ/μ=4, (g) G/μ=4, δ/μ=0, (h) G/μ=4, δ/μ=2, (i) G/μ=4, δ/μ=4. Results are compared with phase velocity angular dependences computed directly using wavefields originating from a line source (blue dotted curve). Theoretically predicted (i.e. ground truth values) phase (solid red curve) and group (solid blue curve) velocity angular distributions are plotted for comparison.

Processed wavefields can now be used to measure the phase velocity of Rayleigh waves because they can be considered plane waves. The results from the correlation method using the proposed spectral unmixing procedure are presented in Fig. 6. As seen, the results of the computation closely resemble the angular dispersion of phase velocity obtained with the line source and are close to theoretically predicted values except for the region with low signal magnitude (around 45 deg). Little deviations in the phase velocity computed using angular spectrum filtration (blue dots in Fig. 6) from their theoretical predictions (red solid curve) are related to the finite width of the filter that was tuned to reasonably match the angular sampling in the experiments.

### 4.2 Experimental Results

**Fig. 7:**
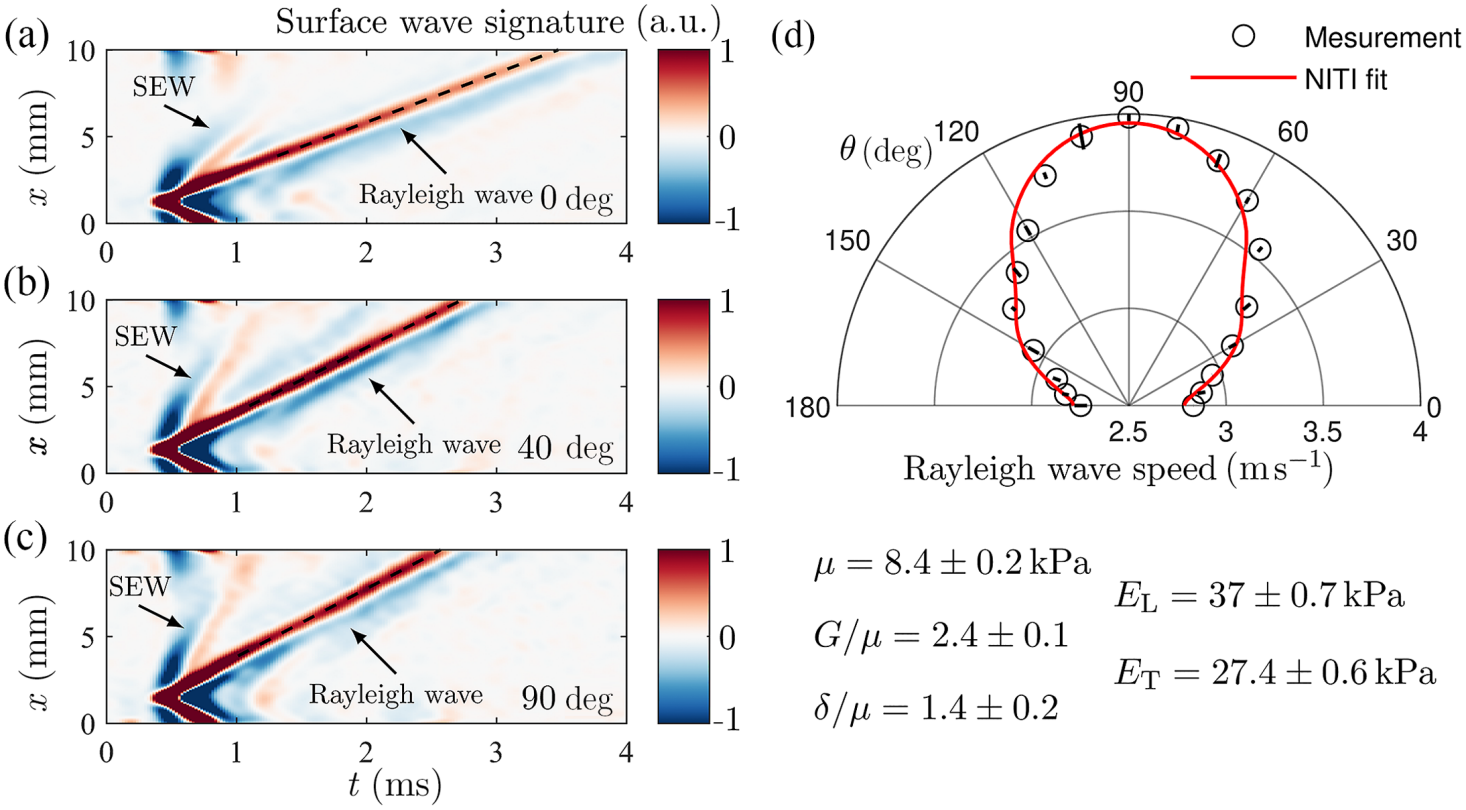
Dynamic OCE measurements with a line A μT source of Rayleigh waves in the anisotropic (NITI) PVA phantom. (a)–(c) Distance-time (XT) plots for different propagation directions: 0, 40, and 90 deg, respectively. Overall, XT plots were recorded for 19 propagation directions (every 10 deg) between forward and backward propagation. (d) Rayleigh-wave angular anisotropy computed using XT plots and reconstructed mechanical moduli of the phantom. The red curve in panel (d) corresponds to fitting experimental data with the NITI model (open circles). The black line in panel (d) corresponds to error bars, computed from the standard deviation over three repeated scans at each location (Video 3, MP4, 9.43 MB [URL: https://doi.org/10.1117/1.JBO.30.12.124503.s3]).

Figures 7(a)–7(c) show the space-time (XT) plots of recorded Rayleigh waves at different directions in the phantom relative to the sample’s symmetry axis (nineteen in total, i.e., recorded for every 10 deg in the range between forward and backward directions). The phase velocity of Rayleigh waves was computed directly from the wavefields using the cross-correlation method.^30^ The phase velocity angular dependence was fit with the NITI model to reconstruct all 3 (G, μ and δ) moduli, as shown in Fig. 7(d). A video of the wave propagation in the phantom following multiple line source excitations is presented in Video 3. This confirms the robustness of the line source excitation technique for selective excitation of spatial harmonics in a NITI material, providing direct access to the mechanical wave phase velocity and straightforward reconstruction of anisotropic elastic moduli. Note that super-shear evanescent waves (SEWs),^13^ propagating faster than the Rayleigh wave, are present in the near field of the source and rapidly decay with distance. SEW speed varies with an angle, which can potentially be used for the reconstruction of elastic moduli. However, the simple relationship between Rayleigh wave and SEW speeds in an isotropic medium, $c_{\mathrm{SEW}}=1.9554\;c_{s}$, does not work for anisotropic media. The solution for SEW in a NITI material is complicated and has not yet been used yet to infer elastic moduli because it requires complex numerical inversion.

## 5 Discussion

Although dynamic OCE techniques are now well developed and can perform tissue scanning in a fully noncontact manner in near real time, reconstruction of tissue mechanical moduli is often not trivial, especially in anisotropic or/and bounded media. Commonly used oversimplified approaches often lead to very inaccurate reconstruction of mechanical moduli, sometimes differing by several orders of magnitude even for very similar samples.^14^^,^^31^^–^^33^ It is worth mentioning that recent approaches, such as torsional wave elastography,^34^ have shown potential for measuring wave speed in thin media (e.g., cornea) while minimizing the effects of guided wave dispersion. However, proper handling of material anisotropy remains crucial regardless of the method used for Rayleigh wave excitation or detection.

In this study, we investigated various approaches to compute the propagation speed of Rayleigh mechanical waves mainly used in dynamic OCE to reconstruct soft tissue elastic moduli. When the frequency dispersion of (i.e., for low viscosity) mechanical waves can be neglected, the propagation speed of Rayleigh waves in bulk isotropic media can be computed using multiple methods. In such cases, the computed group velocity coincides with the phase velocity, and the distinction between the two can be omitted. The computed phase velocity can then be used to reconstruct elastic moduli. Different excitation geometries, i.e., point-like and line excitation sources of Rayleigh waves^5^^,^^6^ creating circular and plane waves, respectively, will yield the same propagation speed. Note, however, that for complicated excitation geometries or when the length of the line source is not large enough compared with the characteristic signal wavelength, diffraction effects may play a role in phase velocity dispersion and must be considered.

The situation is totally different for anisotropic materials, which is the case for most biological tissues (skin, cornea, sclera, heart, muscle, tendon, and even brain). In such cases, the phase and energy flux of waves may propagate in different directions, leading to significant differences between group and phase velocities. Treating the computed group velocity as the phase velocity can result in significant errors in reconstructed tissue elastic constants, which unfortunately is not uncommon in dynamic OCE.

Although many studies have correctly used the group velocity angular anisotropy in conventional shear wave elastography^16^^–^^19^ to compute G and μ, extending this approach to Rayleigh waves is not straightforward. Indeed, the in-plane (XZ-plane) angular dependence of the Rayleigh wave phase velocity is defined by all 3 (G, μ, and δ) shear moduli, whereas in-plane bulk shear wave propagation is determined only by G and μ.^16^^–^^19^ As clearly shown here, the group velocity angular dependence often exhibits focusing and defocusing points, and ambiguity regions (see Figs. 1, 3, 4, and 6 and Video 1). Therefore, relying on group velocity measurements along certain directions can lead to significant inaccuracies.

By contrast, Rayleigh wave phase velocity angular dependence has no ambiguities and can be used directly for moduli reconstruction. The advantage of a line source is direct access to the phase velocity because it launches a plane wave in a specified direction. In addition, unlike spherical waves from a point source, plane waves do not attenuate with propagation distance by diffraction. This means that positions far from the source can be measured with a high signal-to-noise ratio compared with a point-like source. The disadvantage of this approach, however, is that the line source must be rotated to access all propagation directions. This may lead to increased scan time and the possibility of motion artifacts. However, when a large tissue area is scanned, a proper scanning diagram may produce scan times equivalent to those for a point-like source; i.e., instead of changing the position of the point-like source to cover the desired tissue area, angular scanning with the line source can be performed.

In this paper, we also showed that Rayleigh wave fields generated with a point-like source can be reprocessed to access the phase velocity. Figure 6 shows that this approach is equivalent to line source excitation. However, its disadvantage is wave attenuation with distance from the source due to diffraction, which may result in a low signal-to-noise ratio (SNR) of the measured wave speed and consequent highly inaccurate moduli reconstruction. Spatial filtering, a key part of this approach, may further reduce SNR, especially in cases when the spatial resolution of the imaging system is not sufficient. As such, post-processing should be performed with care and in accordance with system limitations. Finally, we also note that the velocity cannot be estimated too close to the source due to super-shear evanescent waves (SEW,^13^) in this region.

Experiments on anisotropic PVA phantoms demonstrated that AμT-OCE with the line excitation of Rayleigh waves can quantify soft tissue anisotropy without ambiguities and with sufficient SNR, which may not be the case for point-like excitation. The spatial harmonic selectivity of the line source makes it a promising approach for measuring tissue pre-stress or tension,^18^^,^^35^ where similar anisotropic wave propagation effects are expected. It is important to note that translation to clinical settings is most often accompanied by reduced SNR and increased artifacts due to tissue heterogeneities, surface irregularities, and curvature effects, as commonly encountered, for example, in skin elasticity studies.^15^ Thus, every effort to improve SNR and robustness will be required for effective clinical translation.

## 6 Conclusion

In this work, we considered the practical case of dynamic OCE utilizing Rayleigh waves in anisotropic biological tissues (e.g., muscle, tendon, skin, and cornea). In particular, we focused on accurate elastic moduli reconstruction from measured wave propagation speeds. In a NITI material, the speed of propagating Rayleigh waves in an arbitrary direction is defined by all 3 (G, μ and δ) mechanical moduli, unlike isotropic tissue where the Rayleigh wave speed is simply equal to $0.955\sqrt{\mu /\rho }$. To reconstruct elastic moduli in anisotropic tissue, the in-plane angular dependence of the Rayleigh wave velocity should be computed from experimental data. Unlike the isotropic case, where the phase and group velocities are the same, there is a tremendous difference between the group and phase velocities of Rayleigh waves in anisotropic media. Using the group velocity in the expressions for the phase velocity is incorrect and can lead to a completely erroneous reconstructed set of moduli. We also showed that, due to ambiguities in the group velocity angular dependence, accurate computation of this parameter from experimental data may be difficult. The phase velocity angular dependence has no ambiguities and can be used to directly estimate moduli. Calculating the phase velocity, however, requires special procedures. In this study, we described two of them. Using a line source for Rayleigh wave excitation directly leads to the phase velocity in the direction normal to the excitation line. For a point-like source, spatial filtering must be performed in the angular spectrum domain prior to velocity calculation. Our study suggests that using the line source is beneficial when a surface area needs to be scanned, whereas a point-like source with proper phase velocity reconstruction is well suited to single point estimation of moduli or when tissue motion is significant.

## 7 Appendix: Supplementary Videos

The following videos were referenced in this article:

Video 1. Anisotropic wave propagation following a point source excitation in a NITI material with μ=10  kPa, G/μ=4, and δ/μ=0, as shown in Fig. 1. The spatial sampling is 10  μm, and the temporal sampling is 5  μs. For three specific directions (0, 45, and 90 deg), where 0 deg corresponds to the axis of symmetry, the detailed spatial profile evolutions are given (MP4, 6.16 MB).
Video 2. Anisotropic wave propagation following multiple line source excitations in a NITI material with μ=10  kPa, G/μ=4, and δ/μ=0, as shown in Fig. 2. For every angle, multiple point sources are recombined to simulate a linear excitation. For each of these angles, the spatio-temporal signature of the wavefield at the center of the plane wave is selected and combined to generate the video. For three specific directions (0, 45, and 90 deg), where 0 deg corresponds to the axis of symmetry, the detailed spatial profile evolutions are given (MP4, 6.11 MB).
Video 3. Anisotropic wave propagation following multiple line source excitations in a NITI phantom, as shown in Fig. 7. For each angle, the spatio-temporal signature of the wavefield at the center of the plane wave is selected and combined to generate the video. For three specific directions (0, 40, and 90 deg), where 0 deg corresponds to the axis of symmetry, the detailed spatial profile evolutions are given (MP4, 9.43 MB).

## Supplementary Material

10.1117/1.JBO.30.12.124503.s01

10.1117/1.JBO.30.12.124503.s1

10.1117/1.JBO.30.12.124503.s2

10.1117/1.JBO.30.12.124503.s3

## Data Availability

All data in support of the findings of this paper are available upon reasonable request.
