# Supplementary material for: Reconstruction of tensile and shear elastic moduli in anisotropic nearly incompressible media using Rayleigh wave phase and group velocities
Source: J Biomed Opt. 2025 Aug 5;30(12):124503. doi: 10.1117/1.JBO.30.12.124503 (PMC12334138; doi:10.1117/1.JBO.30.12.124503)
Supplement: Supplementary file 1 [file JBO_030_124503_SD001.docx]

**Captions for Supplementary Videos**

Supplementary Material 1.

**Video 1.** Anisotropic wave propagation following a point source excitation in a NITI material with $\mu=10 \mathrm{kPa}$, $G/\mu=4$, and $\delta/\mu=0$, as shown in Fig.1 of the main manuscript. The spatial sampling is 10 µm and the temporal sampling is 5 µs. For three specific directions (0°, 45° and 90°), where 0° corresponds to the axis of symmetry, the detailed spatial profile evolutions are given.

Supplementary Material 2.

**Video 2.** of anisotropic wave propagation following multiple line source excitations in a NITI material with $\mu=10 \mathrm{kPa}$, $G/\mu=4$, and $\delta/\mu=0$, as shown in Fig.2 of the main manuscript. For every angle, multiple point sources are recombined in order to simulate a linear excitation. For each of these angles, the spatio-temporal signature of the wavefield at the center of the plane wave is selected and combined to generate the video. For three specific directions (0°, 45° and 90°), where 0° corresponds to the axis of symmetry, the detailed spatial profile evolutions are given.

Supplementary Material 3.

**Video 3.** Anisotropic wave propagation following multiple line source excitations in a NITI phantom, as shown in Fig.7 of the main manuscript. For each angle, the spatio-temporal signature of the wavefield at the center of the plane wave is selected and combined in order to generate the video. For three specific directions (0°, 40° and 90°), where 0° corresponds to the axis of symmetry, the detailed spatial profile evolutions are given.
